# Editorial: Exploring the role, function, and behavior of the anaerobic microbiota: Species, communities, networks, biotechnological potential, and biomedical aspects

**DOI:** 10.3389/fmicb.2023.1128031

**Published:** 2023-02-01

**Authors:** Andreas Otto Wagner, Maria Westerholm, Blaž Stres, Jan Kopečný

**Affiliations:** ^1^Department of Microbiology, Universität Innsbruck, Innsbruck, Austria; ^2^Department of Molecular Sciences, BioCentre, Swedish University of Agricultural Sciences, Uppsala, Sweden; ^3^Department of Catalysis and Chemical Reaction Engineering, National Institute of Chemistry, Ljubljana, Slovenia; ^4^Institute of Animal Physiology and Genetics, Academy of Sciences of the Czech Republic (ASCR), Prague, Czechia

**Keywords:** anaerobic microorganisms, anaerobic microbiota, microbial communities, anaerobic fungi, silage microorganisms, methanogenesis

Microbial degradation of organic matter under anaerobic conditions can be performed *via* so-called alternative electron acceptors and, in habitats lacking these, *via* fermentation. Processing of breakdown products is driven cascade-like by several groups of microorganisms, often interdepending and working together, leading to specialized consortia that are fully able to degrade organic matter. Such natural cycles can be used for biotechnological applications adapting anaerobic processes and transforming them into a controlled environment. Although these systems are usually showing good performance, detailed knowledge of the underlying processes and the engaged microorganisms is still scarce. Many of the applied processes, however, harbor significant future potential for the isolation of new microbial species with probably new, yet undiscovered physiological and/ or genetic potentials.

The Research Topic “*Exploring the role, function, and behavior of the anaerobic microbiota: Species, communities, networks, biotechnological potential, and biomedical aspects*” comprises four original articles and reviews by more than 30 authors on a range of microbial services in various habitats.

Xue et al. isolated anaerobic fungi from the rumen and fecal samples of the Bactrian camel, investigated their potential for lignocellulose bioconversion, and tested cultures for various fermentation and digestion parameters when applying different substrates. The highest lignocellulosic bioconversion potential was found for strains derived from Bactrian camel samples, indicating a potential future source for the isolation of yet uncultured anaerobic fungi that can also possibly be suited for industrial applications.

The article of Gong et al. enriched a fungal community capable of degrading polycyclic aromatic hydrocarbon (PAH) using phenanthrene as the sole carbon source to improve the methanogenic microbiota and its coal-degrading ability. Their results suggested the potential to increase methane production through the application of indigenous PAH-degrading fungi *via* an improved fermentation of coal's aromatics.

For ruminants, *Leymus chinensis* represents an important feed crop. The study by Wu et al. investigated the role of *Lactiplantibacillus plantarum* and *Lentilactobacillus buchneri* during the fermentation of wilted *L. chinensis* silage and evaluated silage quality, aerobic stability, and microbial dynamics. Silage inoculated with *L. plantarum* and *L. buchneri* showed an improved fermentation quality and the addition of these microorganisms inhibited aerobic spoilage in a 7-day spoilage test.

Vinzelj et al. set up a cross-laboratory, year-long study on variability and preservation issues during the cultivation of anaerobic fungi (AF) and evaluated various protocols for anaerobic fungi preservation. They showed a significant impact on handling issues that influenced the variability of results. Moreover, it was demonstrated that biomass preservation in liquid nitrogen resulted in the highest survival rates followed by agar preservation at 39°C.

In summary, the articles included in the Research Topic demonstrate the importance of anaerobic microorganisms in various habitats. The results of such studies emphasize the range and power of microbial services in anaerobic processes but also the possible biotechnological applications in the future.

## Author contributions

AW wrote the manuscript. All authors read and approved the final manuscript.

